# How Population Structure Impacts Genomic Selection Accuracy in Cross-Validation: Implications for Practical Breeding

**DOI:** 10.3389/fpls.2020.592977

**Published:** 2020-12-16

**Authors:** Christian R. Werner, R. Chris Gaynor, Gregor Gorjanc, John M. Hickey, Tobias Kox, Amine Abbadi, Gunhild Leckband, Rod J. Snowdon, Andreas Stahl

**Affiliations:** ^1^The Roslin Institute and Royal (Dick) School of Veterinary Studies, University of Edinburgh, Easter Bush Research Centre, Midlothian, United Kingdom; ^2^NPZ Innovation GmbH, Holtsee, Germany; ^3^German Seed Alliance GmbH, Hohenlieth, Germany; ^4^Department of Plant Breeding, IFZ Research Centre for Biosystems, Land Use and Nutrition, Justus Liebig University, Giessen, Germany; ^5^Julius Kuehn Institute (JKI), Federal Research Centre for Cultivated Plants, Institute for Resistance Research and Stress Tolerance, Quedlinburg, Germany

**Keywords:** predictive breeding, genomic prediction, oilseed rape, nested association mapping population, structure

## Abstract

Over the last two decades, the application of genomic selection has been extensively studied in various crop species, and it has become a common practice to report prediction accuracies using cross validation. However, genomic prediction accuracies obtained from random cross validation can be strongly inflated due to population or family structure, a characteristic shared by many breeding populations. An understanding of the effect of population and family structure on prediction accuracy is essential for the successful application of genomic selection in plant breeding programs. The objective of this study was to make this effect and its implications for practical breeding programs comprehensible for breeders and scientists with a limited background in quantitative genetics and genomic selection theory. We, therefore, compared genomic prediction accuracies obtained from different random cross validation approaches and within-family prediction in three different prediction scenarios. We used a highly structured population of 940 *Brassica napus* hybrids coming from 46 testcross families and two subpopulations. Our demonstrations show how genomic prediction accuracies obtained from among-family predictions in random cross validation and within-family predictions capture different measures of prediction accuracy. While among-family prediction accuracy measures prediction accuracy of both the parent average component and the Mendelian sampling term, within-family prediction only measures how accurately the Mendelian sampling term can be predicted. With this paper we aim to foster a critical approach to different measures of genomic prediction accuracy and a careful analysis of values observed in genomic selection experiments and reported in literature.

## Introduction

Over the last two decades, the application of genomic selection has been extensively studied in various crop species, and it has become a common practice to report prediction accuracies using random cross validation. However, genomic prediction accuracies obtained from random cross validation can be strongly inflated due to population or family structure, a characteristic shared by many experimental plant populations and breeding populations. We think that understanding the concept of prediction accuracy and its determinants in the context of genomic selection is of great importance for the strategic orientation of practical plant breeding programs, where decisions based on imprecise selection parameter estimates or wrong assumptions can pose a high risk to economic efficiency.

In this paper, our intention is to demonstrate and explain why genomic prediction accuracy can only be interpreted in consideration of the prediction scenario, which requires a clear prediction objective, and to show how population and family structure can have an effect on genomic prediction accuracies obtained from different cross validation scenarios.

Genomic selection is a form of marker-assisted selection which utilizes associations between the phenotype and a large number of molecular markers across the whole genome (Goddard and Hayes, 2007). By capturing these associations, genomic selection can enable to predict genotype performance and make selections based on markers even before a seed has been planted. The application of genomic selection involves only a few key steps. Initially, a training population which is both phenotyped and genotyped has to be generated. The training population is used to establish a genomic prediction model that then can be used to predict the genetic values of unphenotyped genotypes or to obtain improved evaluations in case phenotypic information is strongly limited, e.g., in early selection stages. If applied appropriately, breeding strategies using genomic selection can benefit from optimized resource allocation, accurate evaluation of unphenotyped germplasm, and shortened breeding cycle time (Heffner et al., 2010; Heslot et al., 2015).

The rise of genomic selection was largely driven by the continuous advancements in genotyping technology and statistical computing, but especially the availability of user-friendly and open-access software paved the way for the widespread application of genomic selection. The implementation in practical plant breeding, however, is still in the early stages. The cost-effective exploitation of genomic selection does not only depend on the availability of efficient software solutions, but also requires a fundamental understanding of selection theory and the role of genomic selection in that context. While the general goal of genomic selection can simply be described as increasing genetic gain per unit of time compared to phenotypic selection without increasing costs (Crossa et al., 2017), achieving this goal can be challenging. It requires that potential benefits and drawbacks associated with genomic selection are thoroughly compared against each other in the context of a multi-stage breeding program (Heslot et al., 2015). With this in mind, a breeder needs to quantify the impact of a genomic selection strategy on genetic gain per unit of time when alternative breeding programs are to be compared.

The breeder's equation (Lush, 1937) offers a framework to estimate the effect of a selection strategy on genetic gain per unit of time. In terms of a breeding population, genetic gain is described as a directional change in the population mean, which is referred to as response to selection (R). Using the breeder's equation, the expected response to selection in the next generation can be approximated based on four key parameters:

(1) $$\begin{array}{l} R=\frac{i\;\sigma_{G}\;h}{L} \end{array}$$

where *i* is the selection intensity, σ_*G*_ is the amount of genetic variation in the population, *h* represents the accuracy of selection and *L* is the breeding cycle time. The four components are multiplicatively related and selection response can be increased by either increasing the value of the components in the numerator or by decreasing breeding cycle time in the denominator. Genomic selection can be used to effectively address each of the components in the breeder's equation (Hickey et al., 2017). Depending on the crop species and the structure of the breeding program, especially the breeding cycle time, selection intensity and selection accuracy can be directly addressed to increase genetic gain per unit of time.

It is straightforward to quantify how genomic selection could manipulate breeding cycle time and selection intensity compared to a reference breeding program. However, precise estimates of prediction accuracy can only be obtained *a posteriori*, i.e., after the selection process has been completed. Furthermore, prediction accuracy is trait-specific and depends on numerous factors including e.g., the germplasm, the effective population size, the size and relatedness of the training population with the individuals to be predicted, and the quality and number of phenotypic records per individual (Habier et al., 2007; Daetwyler et al., 2008; Hayes, 2008; Goddard, 2009; Pszczola et al., 2012; Hickey et al., 2014; Heslot et al., 2015). However, to assess the value of genomic selection in a multi-stage plant breeding program, reliable estimates of genomic prediction accuracy are vital when compared to phenotypic selection accuracies. Considering that the expected genomic prediction accuracy will have direct implications on selection intensity and breeding cycle time, inflated estimates of prediction accuracy can have adverse effects on the outcome of the selection process.

A common practice to assess genomic prediction accuracy is random cross validation. In random cross validation, a set of individuals that are both genotyped and phenotyped is randomly partitioned into a training population to train the prediction model and a validation population. The phenotypes in the validation population are then predicted using the model, and the correlation between the predicted and observed phenotypes over multiple iterations serves as a measure of genomic prediction accuracy. Random cross validation can be a useful technique for model validation or comparison of different prediction models. Prediction accuracies obtained from random cross validation, however, are specific for the population under consideration and do not necessarily represent the prediction accuracy to be expected in a practical breeding program. This is because population and family structure can have a substantial effect on estimates of prediction accuracy.

Plant breeding populations often exhibit strong population structure due to somewhat diverse genetic backgrounds as well as family structure. Windhausen et al. described already in 2012 the effect of population structure on genomic prediction accuracies obtained from random cross validation. In a diversity set of hybrids grouped into eight breeding populations, they showed that predictive ability mostly resulted from differences in the mean performance of the breeding populations. Other studies have shown that prediction accuracies within and among families can substantially differ in structured populations, including studies that used stochastic simulation (e.g., Hickey et al., 2014) and studies based on real data sets from maize breeding programs (Massman et al., 2013; Lehermeier et al., 2014) and triticale breeding programs (Würschum et al., 2017).

These studies were of great importance to broaden our understanding of genomic selection and its application in plant breeding programs. However, we found that comprehension of the effect of population and family structure on prediction accuracy requires an understanding of the concept and composition of the breeding value. The main objective of this study was to make this effect and its implications for practical breeding programs comprehensible for breeders and scientists with a limited background in quantitative genetics and genomic selection theory. We intend to foster a critical approach to different measures of genomic prediction accuracy and a careful analysis of values observed in genomic selection experiments and reported in literature.

## Materials and Methods

The plant material consisted of 940 *Brassica napus* hybrids from 46 families broadly representing the genetic diversity within the species-wide gene pool. Hybrid genotypes were characterized using a set of 23,857 SNP markers from the Brassica 60k SNP array and genomic predictions were done using a ridge-regression BLUP (rrBLUP). Genomic prediction accuracies were assessed in three different prediction scenarios using random cross validation and within-testcross family validation to demonstrate the effect of population structure and relatedness between the training population and the validation population on genomic prediction accuracy.

### Plant Material

**Figure 1:**
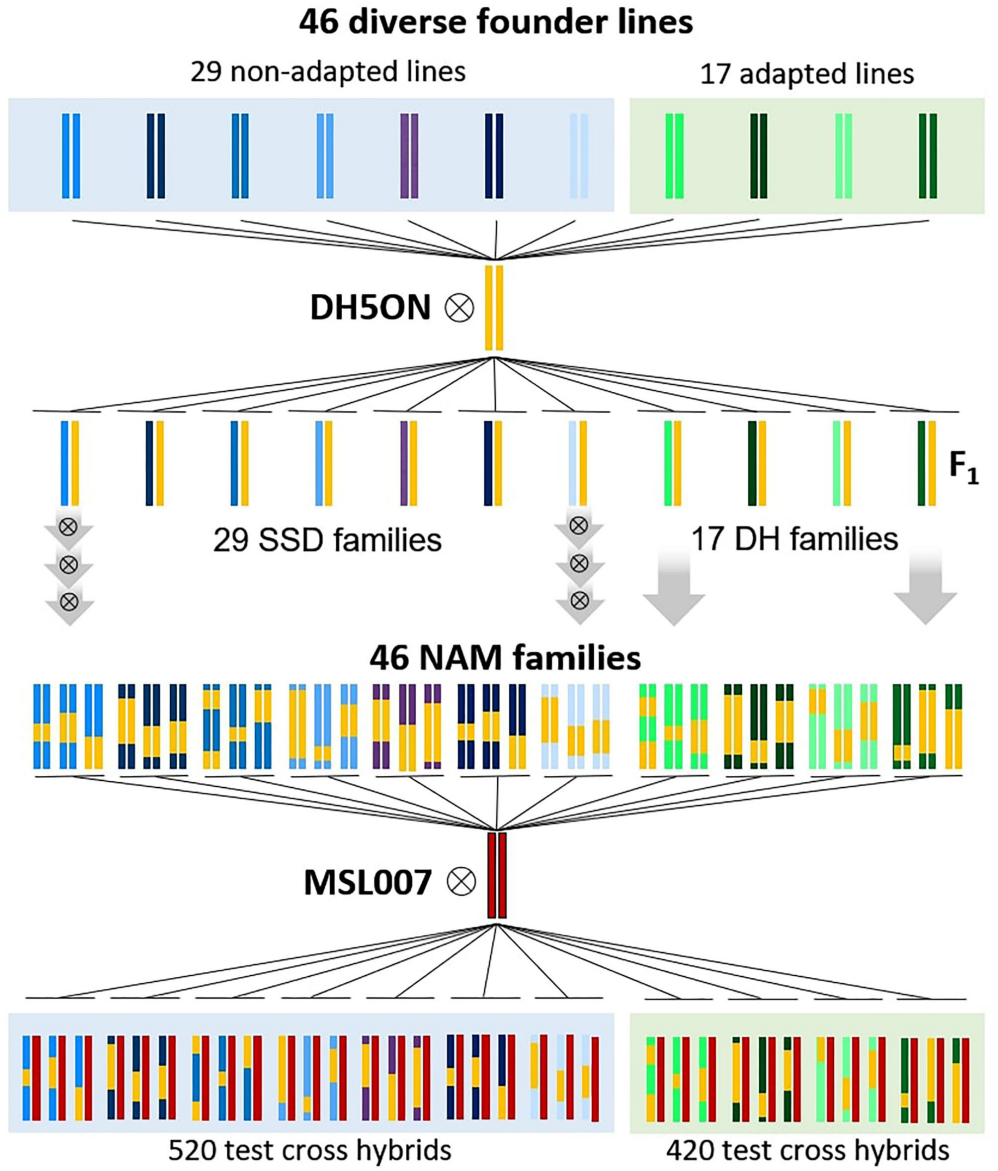
Schematic crossing scheme for development of the *Brassica napus* Nested Association Mapping (BnNAM) population based on 46 founder lines and generation of corresponding test hybrids. 29 non-adapted (include kales, fodder rapes and 19 resynthesized lines) and 17 adapted lines (include a broad set of genetic diverse old European varieties and one resynthesized line) were crossed with the common elite parent “DH5Oase x Nugget” (DH5ON). Based on F2 individuals, 29 families of recombinant inbreed lines were generated via three generations of single seed descend (SSD). Seventeen DH families were produced by using doubled haploid technology. Subsequently, 940 test hybrids were generated by crossing of the elite male-sterile parent “MSL007” with paternal recombinant inbred lines or doubled haploid lines.

The plant material comprised 940 *Brassica napus* F1 testcross hybrids generated by controlled pollination of the elite male-sterile maternal parent “MSL007” with paternal doubled haploid (DH) lines or recombinant inbred lines (RIL). All paternal lines were half-sibs and part of the *B. napus* nested association mapping (BnNAM) population (Snowdon et al., 2015). A schematic illustration of the crossing design is presented in Figure 1. The BnNAM population consists of 60 families that broadly represent the genetic diversity within the species-wide gene pool and comprises adapted winter-type *B. napus* lines along with fodder rapes, kales, old European and Asian *B. napus* forms and 20 resynthesized *B. napus* lines (Girke, 2002; Jesske, 2011). The set used in this study represented a sub-selection of 46 families of the BnNAM population and included 420 DH lines derived from 17 families and 520 RILs derived from 29 families. The DH lines were generated from F2 individuals using doubled haploid technology. The RILs were generated from F2 individuals via three generations of single seed descend (SSD). The number of full-sibs within each family ranged from 10 to 28 (Supplementary Table 1) with an average of 20 full-sibs per family.

We will refer the groups of hybrids derived from the same family as testcross families. The two subsets of testcross families derived from DH lines and RILs will be referred to as DH testcrosses and SSD testcrosses, respectively.

### Phenotypic Data

Phenotypic data on four agronomic traits was collected including seed yield, flowering time, oil concentration in the seed and glucosinolate concentration in the seed. The 420 DH testcrosses were tested at 9–12 locations across Germany in the vegetation period 2013/14 (Supplementary Tables 2, 3). The 520 SSD testcrosses were tested at 11 locations across Germany in the vegetation period 2014/15 (Supplementary Tables 2, 4). All field trials used an augmented randomized design. Plots were sown between August 24 and September 5 in both years at a density of 50 seeds per square meter. Fertilization and plant protection were carried out at local specific intensity. Flowering time was defined as days from Jan 1st. Oil concentration and glucosinolate concentration in the seed were determined using near-infrared reflectance spectroscopy (Tillmann et al., 2000) and adjusted relative to a standard water concentration of 9%. Adjusted entry means were calculated separately for the DH testcrosses and the SSD testcrosses using the following linear mixed model:

(2) $$\begin{array}{l} y_{iklm}=\mu +\alpha_{i}+s_{k}+r_{l\left(s_{k}\right)}+c_{m\left(s_{k}\right)}+e \end{array}$$

with μ being the intercept and α_*i*_ being the adjusted entry mean of the *i*th hybrid, defined as fixed factors. The location effects are represented by *s*_*k*_. Row and column effects within each experimental location are included as *r*_*l*(*s*_*k*_)_ being the *l*th row and *c*_*m*(*s*_*k*_)_ the *m*th column, respectively. Location, row and column were defined as random factors. For each hybrid, *y*_*iklm*_ represents the individual observation of the *i*th hybrid tested in environment *k* at row *l* and column *m*. Residual effects were assumed to be normally distributed with $e\sim N\left(0,\sigma_{e}^{2}\;\right)$.

A one-way ANOVA was carried out for each trait to compare the phenotypic variance within-testcross families to the between-family variance in the complete hybrid set.

### Genotyping

The male-sterile maternal line “MSL007” and all 940 pollinators were genotyped with the Brassica 60k SNP array (Clarke et al., 2016; Mason et al., 2017) using genomic DNA extracted from young leaves. Genotyping was outsourced to the service provider TraitGenetics (Gatersleben, Germany). A filtering for single-copy BLAST hits was conducted based on the *B. napus* reference genome “Darmor-bzh” v8.1 (Bayer et al., 2017). Markers were excluded if their 50 bp SNP probe sequence could not be aligned to a unique physical position on the reference sequence without any mismatches (*E*-value ≤ 7,59E^−17^). In addition, all markers with more than 5% missing values and an expected heterozygosity below 0.1 were removed. Since doubled haploids are expected to produce homozygous signals at all SNP loci, markers that exhibited a heterozygosity >3% in the DH lines were also discarded. The final set of SNP markers comprised 23,857 markers. Hybrid genotypes were extrapolated *in silico* based on the parental marker profiles as described in Werner et al. (2018). An additive genomic relationship matrix was calculated based on the first method of VanRaden (2008) and used in a principle component analysis (PCA) to illustrate population structure among the testcrosses based on the first three principal components. Since all hybrids shared the same pollinator, the PCA represented the population structure among the paternal DH and SSD lines. The PCA was added to the supplementary material (Supplementary Figure 1).

### Genomic Prediction Model

Genomic predictions were done using a ridge-regression BLUP (rrBLUP) to calculate genomic estimated breeding values (GEBVs):

(3) $$\begin{array}{l} y=X\beta +Zg+e \end{array}$$

in which *y* is an n^*^1 vector of adjusted entry means and *n* the number of individuals. *X* is an n^*^p incidence matrix relating fixed effects to individuals, and β is a p^*^1 vector of *p* fixed effects including the overall phenotypic mean and the cultivation year, which was different for the DH testcrosses and the SSD testcrosses. *Z* is an n^*^m design matrix containing the allele dosage at the *m* marker loci, *g* is an m^*^1 vector of *m* allele substitution effects, and *e* is the n^*^1 vector of residual effects. The statistical model underlying the rrBLUP assumes that allele substitution effects and residual effects are normally distributed with $g\sim N\left(0,I\sigma_{g}^{2}\right)$ and $e\sim N\left(0,I\sigma_{e}^{2}\;\right)$.

In terms of a hybrid breeding program, the breeding value of a crossing parent can be estimated from a testcross experiment with one or several representative testers coming from the other crossing pool and is referred to as its general combining ability (GCA). Comparable to the GEBV, a genomic estimated GCA describes a genotype's value as a hybrid crossing parent with genotypes from the other crossing pool based on its marker profile. For the sake of simplicity, we will not differentiate between these two concepts and consistently use the term GEBV which is most common in the genomic selection-related literature. We admit that this might not be entirely accurate, but we think that an in-depth distinction between these two concepts might be confusing for the inexperienced reader and can be neglected for the benefit of easy comprehensibility. Furthermore, there is an ongoing debate about if it is beneficial to consider non-additive genetic effects in genomic prediction models for hybrid performance, so far without consensus. For illustration purposes we used a simple ridge-regression BLUP model with an additive genetic term only.

### Evaluation of Prediction Accuracies

Genomic prediction accuracies were assessed as Pearson correlation between observed and predicted performance using random cross validation and within-testcross family validation. In random cross validation, the data set was randomly divided into a training population and validation population over 100 iterations. The size of the validation population was set to 20, which corresponded to the average number of genotypes per testcross family. In the within-testcross family validation, all genotypes from one testcross family were used as validation population while the remaining 45 testcross families served as training population. This was done for each family, respectively.

Three different prediction scenarios were examined in which either the entire set of 940 hybrids was used, or the two subsets of DH testcrosses and SSD testcrosses were considered individually. The three prediction scenarios are hereinafter described in detail.

#### Prediction Scenario 1

In the prediction scenario 1, all 940 hybrids were used to demonstrate the effects of population structure and family structure on genomic prediction accuracy using two different random cross validation approaches and the within-testcross family validation. In detail, these included

- GEBV cross validation: the hybrids in the validation population were predicted based on their individual marker genotypes. Hybrids were randomly divided into training set and validation set.
- Genotypic parent average cross validation: the hybrids in the validation population were predicted based on the parent average marker genotype. The parent average marker genotype of each testcross family was reconstructed as mean allele dosage at each marker locus across all family members. Hybrids were randomly divided into training population and validation population.
- Within-testcross family validation: the hybrids in the validation population were predicted based on their individual marker genotype. All individuals from one testcross family served as validation population while all other families served as training population.

#### Prediction Scenario 2

In the prediction scenario 2, genomic prediction accuracies were evaluated separately for the two subpopulations of DH testcrosses and SSD testcrosses. Four approaches were used to demonstrate the effect of family structure on prediction accuracy for both subpopulations, respectively.

The first three approaches were the same as in scenario 1, applied within the two subpopulations. The fourth approach also used random cross validation. The four approaches included

- GEBV cross validation.
- Genotypic parent average cross validation.
- Within-testcross family validation.
- Phenotypic parent average cross validation: the hybrids in the validation population were predicted based on the parent average phenotype, which was reconstructed as phenotypic family mean.

Prediction of hybrids based on the phenotypic parent average was done using the following linear model:

(4) $$\begin{array}{l} y=X\beta +e \end{array}$$

where *y* is an n^*^1 vector of adjusted entry means, *X* is an n^*^p matrix assigning individuals to one of the *p* families, β is a p^*^1 vector of phenotypic family means and *e* is an n^*^1 vector of independent and identically distributed residuals. Family means were calculated using members of the training population only and no genomic information was used. This was done to mimic the parent average phenotype of a family.

The phenotypic parent average cross validation approach was only applied within the two subsets, respectively, as they were tested in two different years and the phenotypic entry means were not adjusted for the year effect. In a genomic prediction model, this can be corrected for by including year as a fixed factor. In the phenotypic prediction model, however, the contribution of the year effect and the genetic effects to the phenotypic family means could not be disentangled.

#### Prediction Scenario 3

In the prediction scenario 3, genomic prediction accuracies were evaluated across the two subpopulations. The DH testcrosses from vegetation period 2013/14 were predicted using the SSD testcrosses from vegetation period 2014/15 as training population and vice versa.

## Results

### Phenotypic Variation

**Figure 2:**
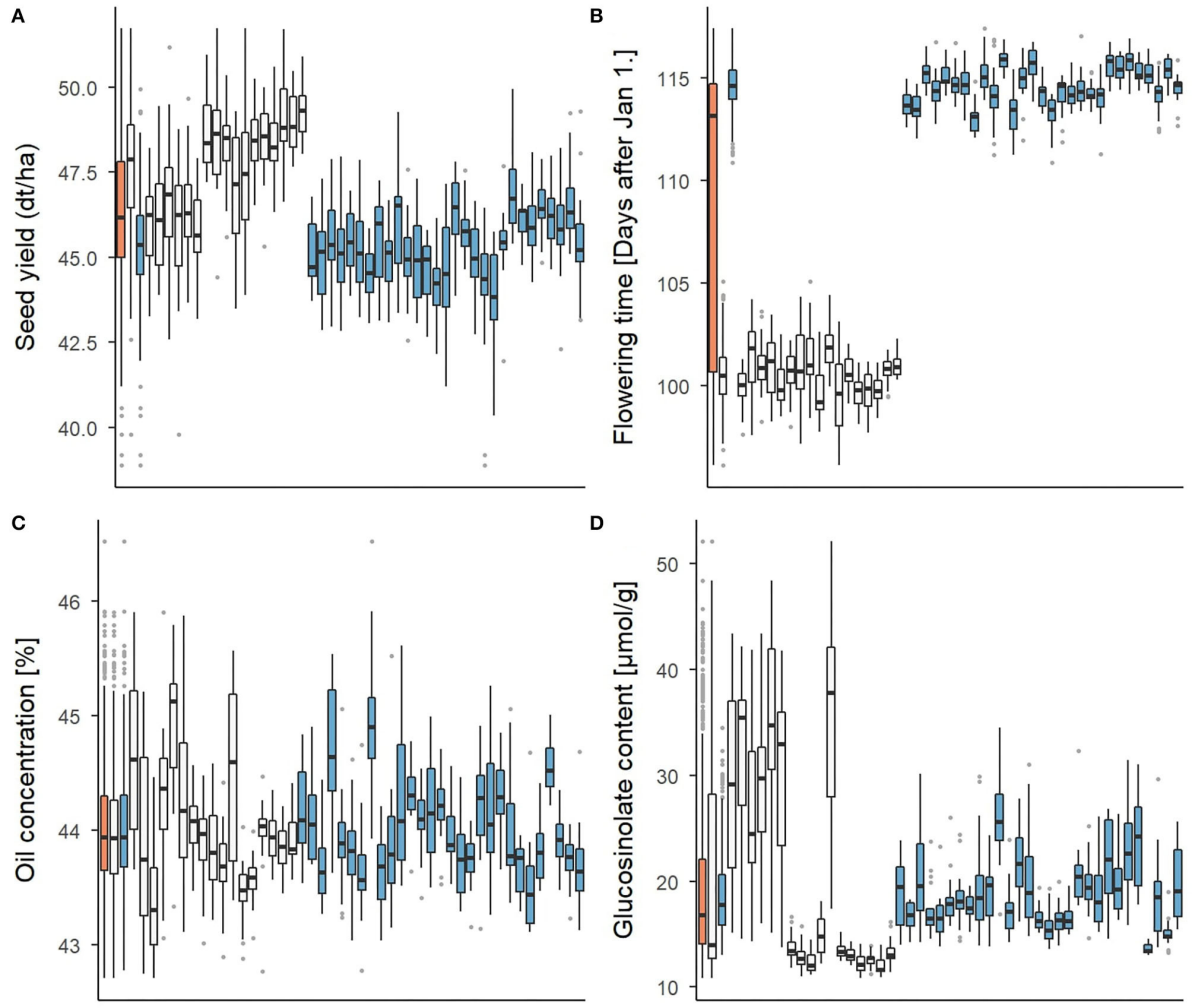
Boxplots representing the phenotypic variation for **(A)** seed yield, **(B)** flowering time, **(C)** oil concentration, and **(D)** glucosinolate concentration. Three columns on the very left show the average of the total set of 940 test hybrids (orange) and of both subsets of DH testcrosses (white) and SSD testcrosses (blue), respectively. Phenotypic distribution within each of the 46 testcross families is presented in the individual boxplots.

The set of 940 testcross hybrids showed extensive phenotypic variation for all four traits. This is shown in Figure 2 for the whole set, the two subsets of DH testcrosses and SSD testcrosses and all 46 families, respectively. Significant (*p* < 0.001) between-family variation was found for all traits in the total set and both subsets using a one-way ANOVA. In what follows, the characterization of the phenotypic variation focusses on the two subsets only, as the mean values of the whole set are slightly skewed toward the SSD testcrosses due to a larger number of individuals. Descriptive statistics of the total set, however, are included in Supplementary Table 5 in addition to those of the DH and SSD testcrosses. The clustering of the complete hybrid set into two subpopulations was further shown using a PCA (Supplementary Figure 1). The first three principal components explained <13% of the variation captured by the markers, which also demonstrates the large genetic diversity of the data set.

For seed yield and glucosinolate concentration, the DH testcrosses showed a higher mean value and a higher standard deviation than the SSD testcrosses. While Figure 2 suggests a relatively continuous variation across families in the SSD testcrosses for the two traits, a clustering into two phenotypically different subgroups is indicated for the DH testcrosses. Glucosinolate concentration in the DH testcrosses showed strong variation with a standard deviation of 10.3 and corresponded to almost three times the standard deviation observed in the SSD testcrosses (4.0). This was mainly driven by seven DH families with very high within-family variation, while the variation in the remaining ten DH families was comparatively low. Flowering started on average 14 days later in the SSD testcrosses than in the DH subset. The variation within the DH and SSD testcrosses was relatively small compared to the variation between the two subsets. Due to this difference, the standard deviation in the total set was 7.1, while the standard deviation in the DH and SSD testcrosses was only 1.4 and 1.1, respectively. For oil concentration, no clear difference could be observed between the DH and SSD testcrosses. Both subsets exhibited a similar mean value (44% oil concentration) and comparable standard deviations of 0.6 (DH crosses) and 0.5 (SSD crosses).

### Prediction Accuracies

For all traits, prediction accuracies obtained from random cross validation were higher than prediction accuracies obtained from the within-testcross family validation. This was observed in the complete set of 940 hybrids and in both subsets of DH testcrosses and SSD testcrosses. In most cases, the GEBV cross validation approach generated the highest prediction accuracies and showed the lowest variation.

#### Prediction Scenario 1

**Figure 3:**
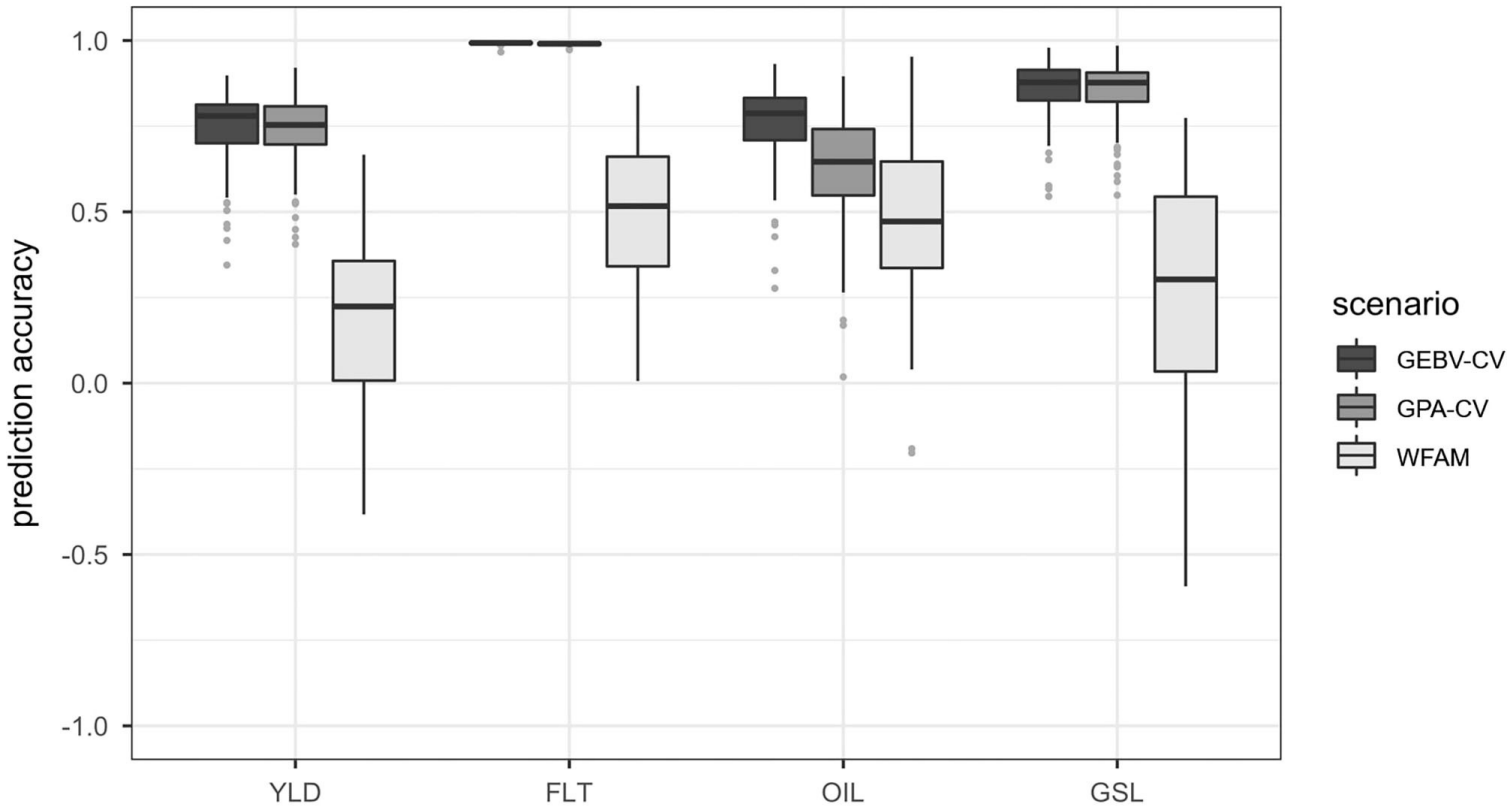
Boxplots representing the prediction accuracies observed in prediction scenario 1 using the total set of 940 testcross hybrids. Traits included seed yield (YLD), flowering time (FLT), oil concentration in the seed (OIL) and glucosinolate content in the seed (GSL). Prediction accuracies were calculated using GEBV cross validation (GEBV-CV), genotypic parent average cross validation (GPA-CV) and within-testcross family validation (WFAM). In the two cross validation approaches, the data set was randomly divided into a training population and validation population over 100 iterations. The size of the validation population was set to 20. In the WFAM, all genotypes from one testcross family were used as validation population while the remaining testcross families served as training population.

The highest prediction accuracies were always observed for the GEBV cross validation approach and the lowest prediction accuracies were observed for the within-family testcross validation. Prediction accuracies for the genotypic parent average cross validation approach were only slightly lower than those of the GEBV cross validation approach for seed yield, flowering time and glucosinolate concentration. A substantial difference between the two cross validation approaches was detected only for oil concentration. The prediction accuracies of prediction scenario 1 are plotted in Figure 3 and summarized in Supplementary Table 6.

Both the GEBV cross validation and the genotypic parent average cross validation approach showed similar variation. Prediction accuracies obtained from the within-testcross family validation were much lower than those from the GEBV cross validation and genotypic parent average cross validation approaches for all traits and exhibited higher variation. Negative prediction accuracies were observed for some of the families in seed yield, glucosinolate concentration and oil concentration. The largest difference between the prediction accuracies from the GEBV cross validation approach and the within-testcross family validation was observed for glucosinolate concentration (0.59). The smallest difference between prediction accuracies was observed for oil concentration (0.28).

#### Prediction Scenario 2

##### DH testcrosses

**Figure 4:**
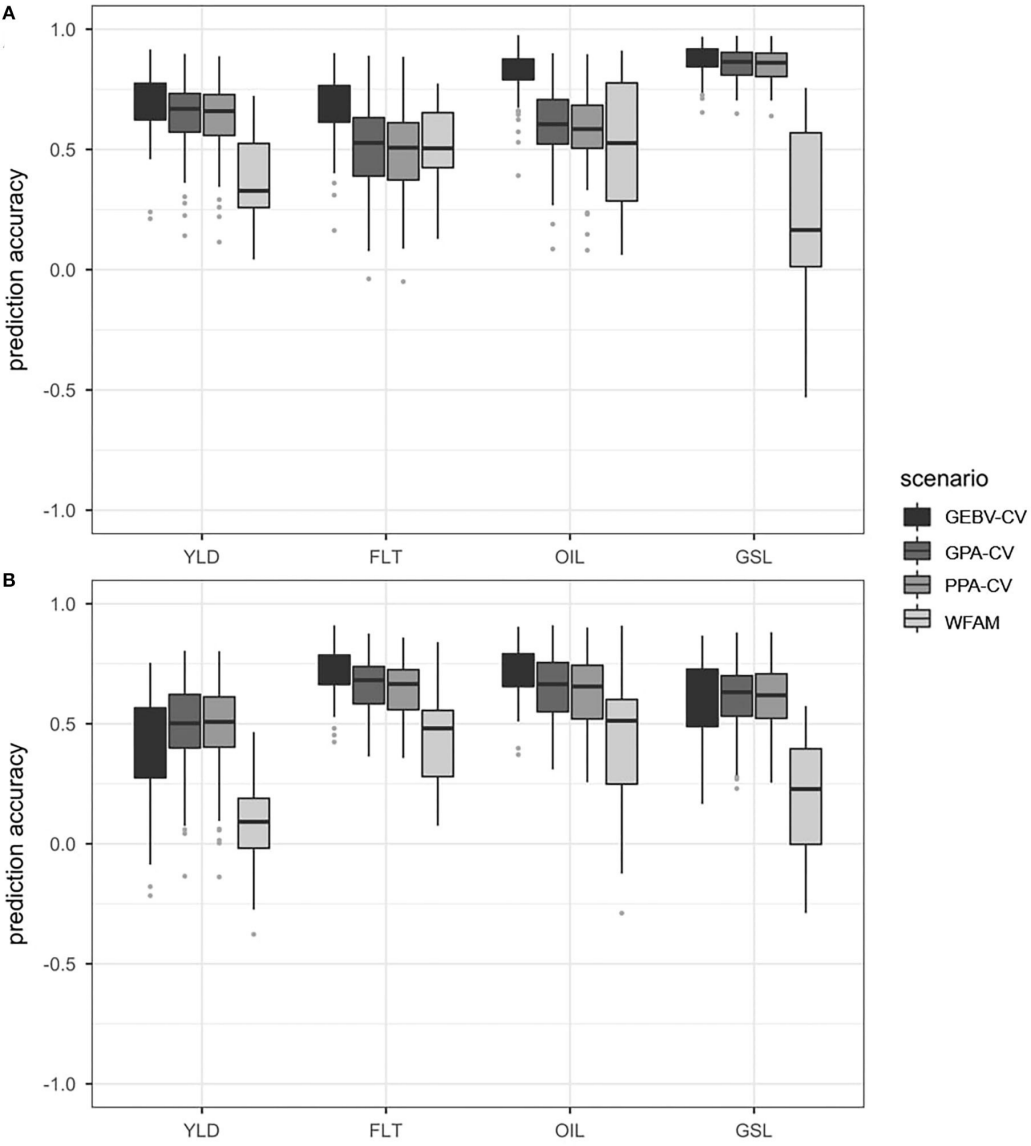
Boxplots representing the prediction accuracies observed in prediction scenario 2 using the DH testcrosses **(A)** and SSD testcrosses **(B)**. Traits included seed yield (YLD), flowering time (FLT), oil concentration in the seed (OIL) and glucosinolate content in the seed (GSL). Prediction accuracies were calculated using GEBV cross validation (GEBV-CV), genotypic parent average cross validation (GPA-CV), phenotypic parent average cross validation (PPA-CV) and within-testcross family validation (WFAM). In the three cross validation approaches, the data set was randomly divided into a training population and validation population over 100 iterations. The size of the validation population was set to 20. In the WFAM, all genotypes from one testcross family were used as validation population while the remaining testcross families served as training population.

In the DH testcrosses, the highest prediction accuracies were always observed for the GEBV cross validation approach, followed by the genotypic parent average cross validation and the phenotypic parent average cross validation approach. The lowest prediction accuracies were always observed for the within-testcross family validation, which showed particularly high variation for oil concentration and glucosinolate concentration. This can be seen in Figure 4A which compares the distribution of prediction accuracies obtained from the four prediction approaches using the DH testcrosses. Average prediction accuracies are shown in Supplementary Table 7.

Prediction accuracies from the genotypic parent average cross validation and the phenotypic parent average cross validation approach were relatively similar across all four traits. For seed yield and glucosinolate concentration, both approaches showed prediction accuracies only slightly below those of the GEBV-cross validation approach. For flowering time and oil concentration, however, prediction accuracies from the genotypic parent average cross validation and the phenotypic parent average cross validation approach were only slightly better than those observed for the within-testcross family validation. Both the genotypic parent average and the phenotypic parent average cross validation approaches also showed higher variation for flowering time than the within-testcross family validation.

While the highest prediction accuracies from the three cross validation approaches were observed for glucosinolate concentration, they were the lowest for the within-family validation, with 8 of 29 families (28%) showing negative values. The difference between the prediction accuracy of the GEBV cross validation approach and the within-family validation was 0.70 for glucosinolate concentration. The lowest difference between the prediction accuracies of the GEBV cross validation approach and the within-family validation was 0.25 in flowering time.

##### SSD testcrosses

In the SSD testcrosses, the GEBV cross validation approach showed the highest prediction accuracies for flowering time and oil concentration, followed by the genotypic parent average and the phenotypic parent average cross validation approach. In seed yield and glucosinolate concentration, however, the GEBV cross validation approach was outperformed by both the genotypic parent average and the phenotypic parent average cross validation approach. For all traits, prediction accuracies observed for the within-testcross family validation were much lower than the prediction accuracies observed for the three random cross validation approaches. This can be seen in Figure 4B which compares the distribution of prediction accuracies obtained from the four prediction approaches using the SSD testcrosses. Average prediction accuracies are shown in Supplementary Table 7.

The within-testcross family validation showed the highest standard deviations for prediction accuracy in all traits except for seed yield, where the GEBV cross validation approach showed the highest standard deviation. Negative prediction accuracies within testcross families were observed in seed yield (8 families), glucosinolate concentration (8 families), and oil concentration (2 families). The highest difference between the average prediction accuracies from the best and worst performing approach was observed for glucosinolate concentration between the genotypic parent average cross validation approach and the within-testcross family validation (0.44). The lowest difference between average prediction accuracies from the best and worst performing approach was measured in flowering time between the GEBV cross validation approach and the within-testcross family validation (0.28).

#### Prediction Scenario 3

Genomic prediction accuracies across the two subsets of DH and SSD testcrosses are presented in Table 1. Prediction accuracies for the DH testcrosses using the SSD testcrosses as training population were higher for seed yield, oil concentration and glucosinolate concentration than vice versa, but lower for flowering time.

**Table 1:** Average prediction accuracies across two subsets in the prediction scenario 3.

	**YLD**	**FLT**	**OIL**	**GSL**
SSD to DH	0.22	0.19	0.54	0.67
DH to SSD	0.17	0.21	0.35	0.33

## Discussion

Genomic prediction accuracies obtained from random cross validation can be strongly inflated due to population and family structure. Estimates of prediction accuracy are specific for the population under consideration and do not necessarily represent the prediction accuracy to be expected in a practical breeding program. Considering that genomic prediction accuracy will have direct implications on setting selection intensity and breeding cycle time in a breeding program, inflated estimates of prediction accuracy can have adverse effects on the outcome of the selection process.

After a brief review of the concept of breeding value, three different prediction scenarios are used to demonstrate how genomic prediction accuracies obtained from among-family predictions and within-family predictions capture different measures of prediction accuracy. We conclude with general guidelines to aid the evaluation of genomic prediction accuracies observed in genomic selection experiments and reported in literature.

### Composition of the Breeding Value

The breeding value of an individual *g*_*o*_ consists of three components:

- half the breeding value of the male parent
- half the breeding value of the female parent, and
- the Mendelian sampling term

(5) $$\begin{array}{l} g_{o}=\frac{1}{2}g_{s}+\frac{1}{2}g_{d}+g_{m} \end{array}$$

where *g*_*s*_ and *g*_*d*_ are the breeding values of the male and female parent, respectively (from animal breeding terminology “sire” and “dam”), and *g*_*m*_ is the Mendelian sampling term. The sum of half the breeding value of the male parent and half the breeding value of the female parent is also often referred to as parent average component. It represents the fact that each parent passes on half of its genes to the next generation. The expectation of the breeding value of a large offspring population is therefore equal to the average of the breeding values of the parents:

(6) $$\begin{array}{l} E\left(g_{o}\right)=\frac{1}{2}g_{s}+\frac{1}{2}g_{d} \end{array}$$

This also implies that the expectation of the Mendelian sampling term over a large number of offspring is zero. For any particular individual, however, *g*_*m*_ has a value that will most likely be non-zero and make the individual's breeding value deviate from the parent average. The Mendelian sampling term reflects the fact that the alleles which are transmitted from a parent to its offspring are sampled at random (Dekkers et al., 2004; Mrode, 2005). As a consequence of the breeding value composition, all full sibs share the same parent average component but differ in their Mendelian sampling term (unless both parents represent the same genotype).

### Prediction of the Breeding Value

Considering that an individual's breeding value consists of half the male parent's breeding value, half the female parent's breeding value and the Mendelian sampling term, we now can address a fundamental question that has to be asked when evaluating prediction accuracy: which kind of prediction accuracy do the cross validation approaches and the within-testcross family validation measure, and how do they differ?

In the cross validation approaches used in prediction scenario 1 and prediction scenario 2, the validation population consisted of genotypes belonging to different families. Therefore, the prediction of an individual's GEBV included two steps (Daetwyler et al., 2007):

- prediction of the parent average component, and
- prediction of the Mendelian sampling term.

In the within-testcross family validation, the validation population consisted of genotypes belonging to the same family, i.e., they were full sibs and shared the same parent average component. Hence, the prediction of the breeding value with reference to all other individuals in the validation population was based only on the Mendelian sampling term.

In summary, this means that the GEBV cross validation and the genotypic parent average and phenotypic parent average cross validation approaches measured how accurately all three components of the breeding value were predicted, while the within-testcross family validation only measured how accurately the Mendelian sampling term was predicted.

### Prediction Scenario 1

In prediction scenario 1, three prediction approaches including GEBV cross validation, genotypic parent average cross validation and within-family validation were compared in the set of 940 rapeseed hybrids that derived from 46 families. The data set was originally generated to broadly represent genetic diversity in the *B. napus* gene pool. We used the complete data set to demonstrate the effect of family structure on prediction accuracy, the effect of population structure on prediction accuracy and the effect of the training population on within-family prediction accuracy. We furthermore show how among-family and within-family prediction capture different measures of prediction accuracy.

#### The Effect of Family Structure on Prediction Accuracy

A combination of two factors strongly contributed to the high prediction accuracies observed in the GEBV cross validation approach:

- there was extensive genetic and phenotypic between-family variation in the total set of testcross hybrids.
- the genotypes in the validation population always had several full sibs in the training population.

As a consequence of the extensive between-family variation in the training population on the one hand and close relatedness between individuals from the same families in the validation population and the training population on the other hand, the genomic prediction model accurately predicted the parent average component (or family membership) of the genotypes in the validation population. This can be concluded from a comparison of the prediction accuracies observed for the GEBV cross validation approach, the genotypic parent average cross validation approach and the within-testcross family validation (Figure 3). While the prediction accuracy of the genotypic parent average cross validation approach was almost as high as the prediction accuracy of the GEBV cross validation approach, accuracies for prediction of only the Mendelian sampling term in the within-testcross family validation were on average low and often negative.

#### The Effect of Population Structure on Prediction Accuracy

The high prediction accuracies in both the GEBV cross validation and the genotypic parent average cross validation approach furthermore were a result of the strong population structure in the hybrid population. The population consisted of two phenotypically different subpopulations which we referred to as DH testcrosses and the SSD testcrosses. The DH testcrosses included adapted material from European Canola breeding programs. The SSD testcrosses consisted of exotic germplasm and resyntheses between *Brassica oleracea* and *Brassica rapa*, which in general do not comply with canola standards and breeding targets (Hasan et al., 2008).

The extensive between-population variation between the DH and SSD testcrosses became visible especially for flowering time, where there was relatively little phenotypic variation within the two subpopulations compared to the large difference between the two subpopulations. Prediction accuracies of both the GEBV cross validation and the genotypic parent average cross validation approaches were almost unity due to accurate subpopulation assignment of the genotypes in the validation population.

For oil concentration, on the other hand, prediction accuracies observed for the genotypic parent average cross validation approach ranged somewhere in between the GEBV cross validation approach and the within-family validation. Means and standard deviations for oil concentration were similar in two subpopulations (Supplementary Table 5) which inhibited a clear grouping of the DH and SSD testcrosses based on their phenotype. As a result, prediction accuracy for oil concentration in the genotypic parent average cross validation was less affected by correct subpopulation assignment than prediction accuracy for the other traits.

#### The Effect of the Training Population on Within-Family Prediction Accuracy

Although the average prediction accuracies observed in the within-family validation were always the lowest for all traits, the within-family validation showed the largest dispersion of prediction accuracies. For oil concentration, the highest within-testcross family prediction accuracy was even higher than the highest prediction accuracy observed for the GEBV cross validation and the genotypic parent average cross validation approach.

Within-family prediction accuracy strongly depends on the suitability of the training population, which should be both closely related to predicted genotypes and as diverse as possible. Close relatedness ensures that the linkage phase between markers and QTL in the training population and the predicted genotypes is similar. High diversity, on the other hand, ensures that many different haplotype combinations are assessed in the training population.

The large dispersion of the within-testcross family prediction accuracies is the result of the huge diversity in relatedness among the 46 testcross families in the total hybrid set. While some families had a large, closely related proportion of individuals in the training population so that the Mendelian sampling term could be accurately predicted, other families were so distantly related to the training population that prediction accuracies were even negative.

#### Among-Family and Within-Family Prediction Accuracy Capture Different Measures of Prediction Accuracy

Prediction accuracies obtained from among-family predictions and within-family predictions capture different measures of prediction accuracy. While among-family prediction accuracy measures how accurately both the parent average component and the Mendelian sampling term can be predicted, within-family prediction only measure how accurately the Mendelian sampling term can be predicted.

**Figure 5:**
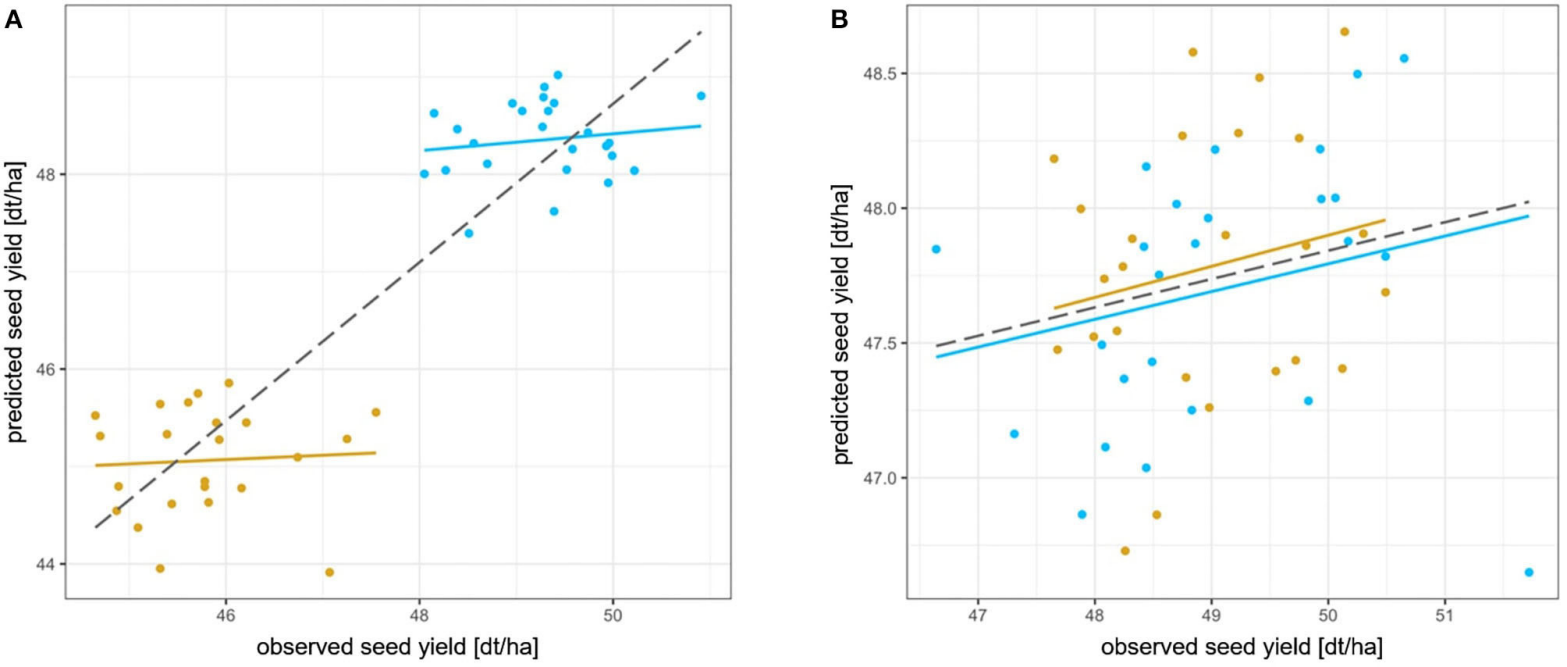
Correlations between observed and genomic predicted seed yield for two testcross families with very different phenotypic performance **(A)** and very similar phenotypic performance **(B)**. The correlation coefficient was used as a measure for prediction accuracy. Prediction accuracies were calculated as within-family prediction accuracies for each of the two families individually (blue and yellow solid lines) and among-family prediction accuracy for both families simultaneously (gray dashed line). The genomic prediction model was trained using all remaining testcross families.

This is further illustrated in Figure 5, which plots the correlation between observed and predicted seed yield for two families with

- very different phenotypic performance (Figure 5A), and
- very similar phenotypic performance (Figure 5B),

while the remaining 44 families were used to train the prediction model.

Prediction accuracy was calculated using two approaches:

- for each of the two families individually.
- for both families simultaneously.

The first approach measures prediction accuracy for the Mendelian sampling term. The second approach measures prediction accuracy for the total breeding value including the parent average component and the Mendelian sampling term.

In Figure 5A, the correlation between observed and predicted seed yield is shown for the two phenotypically very different families. While the within-testcross family prediction accuracies were 0.06 and 0.15, respectively, the overall prediction accuracy was 0.90. This demonstrates how the prediction accuracy was largely driven by the parent average component of the two different families.

In Figure 5B, the correlation between observed and predicted seed yield is shown for the two phenotypically very similar families. Within-testcross family prediction accuracies and overall prediction accuracy were similar due to a similar parent average component of the two families.

### Prediction Scenario 2

In the prediction scenario 2, four prediction approaches were compared in the DH and SSD testcross subpopulations, respectively. The four prediction approaches included the GEBV cross validation, the genotypic parent average cross validation and the within-family validation used already in prediction scenario 1. Furthermore, the phenotypic parent average cross validation approach was added. We used the two subpopulations to demonstrate how prediction accuracies within the two subpopulations decreased for some traits due to reduced population structure, and why the phenotypic parent average cross validation approach often gave prediction accuracies that were comparable to the prediction accuracies obtained from the GEBV cross validation approach.

#### Prediction Accuracy Within the DH and SSD Testcross Subpopulations

In some of the traits, the prediction accuracies observed for the GEBV cross validation and the genotypic parent average cross validation approach were substantially lower within the DH and SSD testcross subpopulations compared to the whole data set. In particular, this became apparent for flowering time, where the phenotypic variation between the two subpopulations was much higher than the variation within the subpopulations. Prediction accuracies of the GEBV cross validation and the genotypic parent average cross validation did not benefit anymore from the correct assignment to one of the two phenotypically different subpopulations.

#### Prediction Accuracies Obtained From the Phenotypic Parent Average Cross Validation Approach

The prediction accuracies observed for the phenotypic parent average cross validation approach were usually similar to those observed for the genotypic parent average cross validation approach, and often almost as high as the prediction accuracies of the GEBV cross validation approach.

The phenotypic parent average cross validation approach can be considered as a simple example of a pedigree-based prediction approach. The offspring phenotypes were used to reconstruct the parent average component of the breeding value, which was used to predict individual performance. Similar to genomic prediction of BLUPs, traditional pedigree-based prediction of BLUPs makes use of information on ancestors and collateral relatives to increase prediction accuracy by adding precision to the parent average component. The Mendelian sampling term of an individual, however, can only be modeled by using its own phenotypic records and progeny information in a pedigree-based BLUP (Daetwyler, 2009).

In contrast, genomic selection enables the prediction of the differences in performance of the Mendelian sampling term based on differences in marker genotypes. It has been shown that genomic selection can increase prediction accuracy compared to traditional pedigree prediction (Hayes et al., 2009; Heslot et al., 2015). However, the advantage of genomic predicted BLUPs over pedigree-based BLUPs can only be realized if a suitable training population is used.

## Conclusions

Genomic prediction accuracies obtained from cross validation can be strongly inflated due to population or family structure and do not necessarily represent accuracies to be expected in a plant breeding program. The breeding value of an individual consists of the parent average component and the Mendelian sampling term. In structured populations, however, the parent average component of the breeding value can often be accurately predicted based on phenotypic information on the parents and does not necessarily require genomic selection. The major advantage of genomic selection to increase prediction accuracy in plant breeding programs is the ability to predict the Mendelian sampling term, which enables to predict the best individuals within a cross. The genomic prediction accuracy of the Mendelian sampling term, however, will be much lower than the genomic prediction accuracy of the total breeding value. This needs to be taken into account when assessing observed or reported genomic prediction accuracies.

An additional problem with the prediction approaches used in the prediction scenarios 1 and 2 is that the effect of genotype by environment interaction (GxE) across multiple year was not considered. However, GxE interaction will have an adverse effect on prediction accuracy from one year to the next, especially when the training set is small or comprises data from only one or a few experimental years. A practically useful approach to evaluate prediction accuracy is prediction across years. In prediction scenario 3, the DH testcrosses from the first experimental year were predicted using the SSD testcrosses from the second year as training population and vice versa (Table 1). Admittedly, there is limited meaning in predicting across two genetically very different data sets. However, it is likely that the low prediction accuracies observed in the third prediction scenario are not only a result of the distant relationship between the two subsets, but also of the effect of different GxE interaction in the two consecutive years.

To aid the evaluation of genomic prediction accuracies, we propose three general guidelines derived from our observations and inspired by Windhausen et al. (2012):

1. Analyse the structure of the data set first and take population and family structure into account when interpreting genomic prediction accuracies. If a data set includes for example exotic or historic genotypes, genomic prediction accuracies observed in this data set might not be representative for a breeding population in a commercial plant breeding program.
2. Clarify the intention behind a prediction experiment. Is the intention to compare the performance of different prediction models, or to estimate genomic prediction accuracies? Which components of the breeding value are to be predicted and in which context is this important? Also compare genomic prediction accuracies to pedigree-based prediction accuracies.
3. Optimally, prediction accuracies are calculated across years to capture the uncertainty imposed by GxE interaction. This comes closest to a realistic prediction scenario in a breeding program and might be useful as rough estimate when considering the impact of GS on the breeder's equation.

## Data Availability Statement

The raw data supporting the conclusions of this article will be made available by the authors, without undue reservation.

## Author Contributions

CW, AS, and RS conceived the study. AA, TK, and GL provided the genotypic and field experimental data. AS analyzed the phenotypic data. CW designed the computational framework and conducted the genomic prediction. RG, GG, and JH contributed to the development of the prediction scenarios. CW and AS wrote the manuscript with further input from RS. All authors contributed to the article and approved the submitted version.

## Conflict of Interest

AA and TK were employed by the company NPZ Innovation GmbH. GL was employed by the company German Seed Alliance GmbH. The remaining authors declare that the research was conducted in the absence of any commercial or financial relationships that could be construed as a potential conflict of interest.
