# Supplementary material for: How Population Structure Impacts Genomic Selection Accuracy in Cross-Validation: Implications for Practical Breeding
Source: Front Plant Sci. 2020 Dec 16;11:592977. doi: 10.3389/fpls.2020.592977 (PMC7772221; doi:10.3389/fpls.2020.592977)
Supplement: Supplementary file 1 [file Data_Sheet_1.PDF]

SUPPLEMENTARIES

How population structure impacts genomic selection accuracy in cross-validation: Implications for practical breeding

Christian R. Werner[1], R. Chris Gaynor[1], Gregor Gorjanc[1], John M. Hickey[1], Tobias Kox[2], Amine Abbadi[2], Gunhild Leckband[3], Rod J. Snowdon[4] and Andreas Stahl[4,5]

[1] The Roslin Institute and Royal (Dick) School of Veterinary Studies, University of Edinburgh, Easter Bush Research Centre, Midlothian EH25 9RG, UK

[2] NPZ Innovation GmbH, Hohenlieth-Hof, Holtsee, 24363, Germany

[3] German Seed Alliance GmbH, Hohenlieth 24363, Germany

[4] Department of Plant Breeding, IFZ Research Centre for Biosystems, Land Use and Nutrition, Justus Liebig University, Heinrich Buff-Ring 26-32, 35392 Giessen, Germany

[5] Julius Kuehn Institute (JKI), Federal Research Centre for Cultivated Plants, Institute for Resistance Research and Stress Tolerance, Erwin-Baur-Strasse 27, 06484 Quedlinburg, Germany

Correspondence:
Andreas Stahl
Julius Kuehn Institute (JKI),
Federal Research Centre for Cultivated Plants,
Institute for Resistance Research and Stress Tolerance,
Erwin-Baur-Strasse 27,
06484 Quedlinburg, Germany
E-Mail: andreas.stahl@julius-kuehn.de
Phone: +49 (0)3946 47 601
Fax: +49 (0)3946 47 600

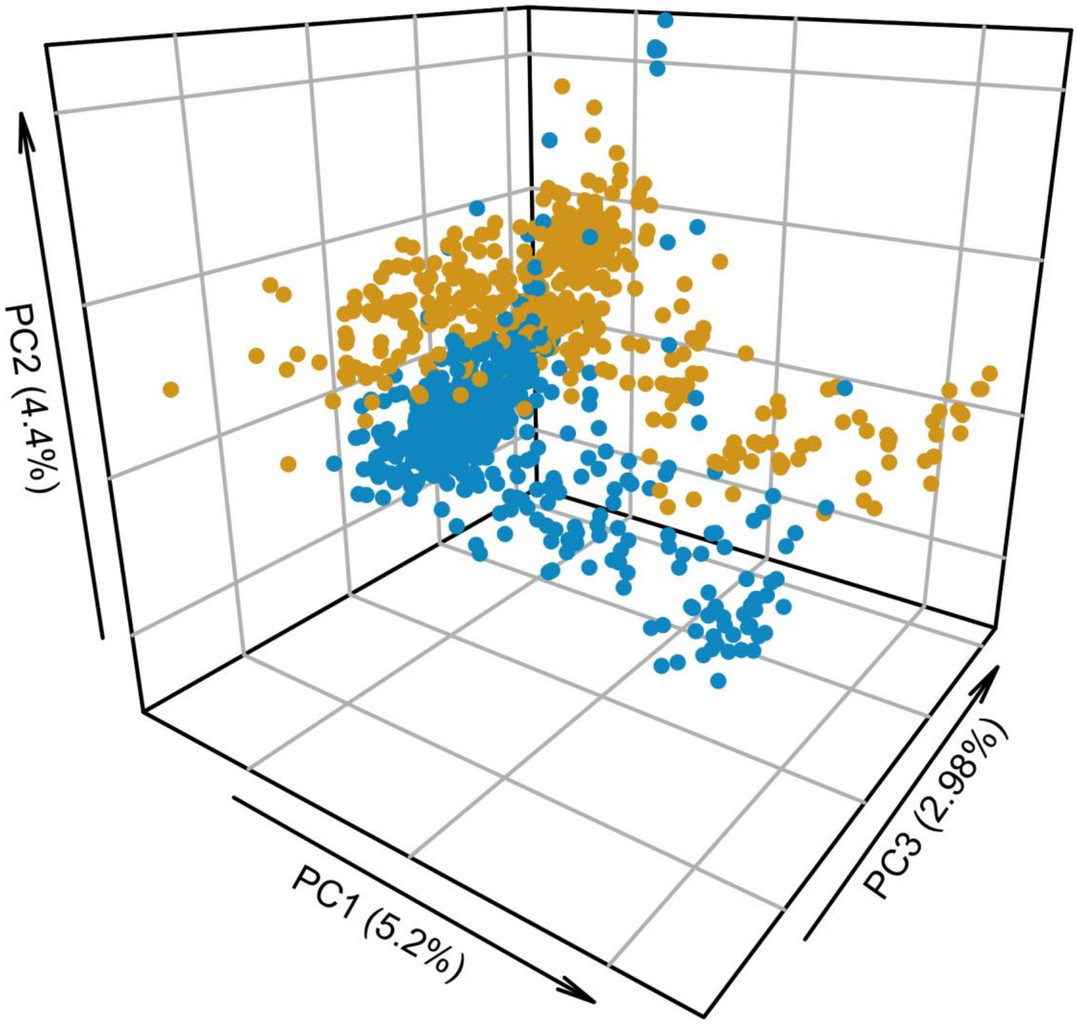

Supplementary-Fig 1 Population structure among the total set of 940 testcross hybrids, represented
by the first three eigenvectors from a principal component analysis (PCA) applied on the additive genomic relationship matrix. The DH testcrosses (yellow) consisted of 420 genotypes, the SSD testcrosses consisted of 520 genotypes. Since all hybrids shared the same pollinator, the PCA represents the population structure among the paternal DH and SSD lines.

Supplementary Tab 1 Family-names and family size (number of full-sibs).

Family No.	Family size	Subset
001	25	DH
002	22	DH
003	25	DH
004	23	DH
007	24	DH
010	23	DH
012	25	DH
015	27	DH
017	25	DH
018	26	DH
021	26	DH
022	24	DH
023	25	DH
024	25	DH
025	25	DH
026	25	DH
029	25	DH
031	17	SSD
032	17	SSD
033	20	SSD
034	18	SSD
035	19	SSD
036	19	SSD
037	18	SSD
038	15	SSD
039	19	SSD
040	20	SSD
041	20	SSD
043	17	SSD
044	18	SSD
045	20	SSD
046	19	SSD
047	16	SSD
048	23	SSD
049	16	SSD
050	18	SSD
051	17	SSD
052	17	SSD
053	12	SSD
054	10	SSD
055	13	SSD
056	13	SSD
057	17	SSD
058	21	SSD
059	28	SSD
060	23	SSD

Supplementary Tab 2 Location of field experiments

Location	Year of harvest	GPS coordinate
Asendorf	2014	52.75722, 9.050262
	2015	52.763541, 8.993924
Bad Salzuflen I	2014	52.090, 8.688333
	2015	52.079715, 8.083141
Bad Salzuflen II	2014	52.066666, 8.75
	2015	52.016666, 8.783333
Einbeck	2014	51.624155, 9.889601
	2015	51.787262, 9.830840
Granskevitz	2014	54.531111, 13.232222
	2015	54.5336111, 13.2327777
Lauenau	2014	52.240186, 9.365333
	2015	52.291698, 9.362589
Leutewitz	2014	51.178357, 13.373945
	2015	51.156024, 13.389899
Lundsgaard	2014	54.795, 9.638055
	2015	54.795, 9.638055
Moosburg	2014	48.501561, 11.940564
	2015	48.501154, 11.936494
Rosenthal	2014	52.294183, 10.097050
	2015	52.305751, 10.167810
Seligenstadt	2014	49.838301, 10.094284
	2015	49.841053, 10.109462
Ziesendorf	2014	53.995, 12.038055
	2015	53.995, 12.038055

Supplementary Tab 3 Overview of collected phenotypic data for seed yield (YLD), flowering time (FLT), oil concentration in the seed (OIL) and glucosinolate content in the seed (GSL) of DH-Hybrids in vegetation period 2013-2014. Sampled locations are indicated with an “x”.

Location	YLD	FLT	OIL	GSL
Asendorf		x	x	x
Bad Salzuflen I	x	x	x	x
Bad Salzuflen II	x		x	x
Einbeck	x	x	x	x
Granskevitz	x	x	x	x
Lauenau	x	x	x	x
Leutewitz	x	x	x	x
Lundsgaard	x	x	x	x
Moosburg	x	x	x	x
Rosenthal	x	x	x	x
Seligenstadt	x	x	x	x
Ziesendorf	x		x	x

Supplementary Tab 4 Overview of collected phenotypic data for seed yield (YLD), flowering time (FLT), oil concentration in the seed (OIL) and glucosinolate content in the seed (GSL) of SSD-Hybrids in vegetation period 2014-2015. Sampled locations are indicated with an “x”.

Location	YLD	FLT	OIL	GSL
Asendorf	x	x	x	x
Bad Salzuflen I	x	x	x	x
Bad Salzuflen II	x		x	x
Einbeck	x	x	x	x
Lauenau	x	x	x	x
Leutewitz	x	x	x	x
Lundsgaard	x	x	x	x
Moosburg	x	x	x	x
Rosenthal	x	x	x	x
Seligenstadt	x	x	x	x
Ziesendorf	x		x	x

Supplementary Tab 5 Descriptive statistics for the adjusted means for seed yield (YLD), flowering time (FLT), oil concentration in the seed (OIL) and glucosinolate content in the seed (GSL).

Sub-population	Parameter	YLD [dt/ha]	FLT [days of year]	OIL [% at 91% dry matter]	GSL [µmol/g]
DH	Mean	47.64	100.54	44.01	20.07
	Max	51.73	105.10	45.90	52.08
	Min	39.80	96.12	42.71	10.83
	Range	11.93	8.98	3.19	41.25
	SD	1.80	1.39	0.62	10.30
	CoV	0.04	0.01	0.01	0.51
RIL	Mean	45.33	114.60	44.02	18.76
	Max	49.95	117.40	46.52	34.49
	Min	38.88	110.89	42.78	12.99
	Range	11.07	6.51	3.74	21.50
	SD	1.40	1.11	0.50	3.96
	CoV	0.03	0.01	0.01	0.21
All	Mean	46.37	108.32	44.01	19.35
	Max	51.73	117.40	46.52	52.08
	Min	38.88	96.12	42.71	10.83
	Range	12.85	21.28	3.81	41.25
	SD	1.96	7.10	0.56	7.51
	CoV	0.04	0.07	0.01	0.39

Mean: Arithmetic mean, Min: Lowest value of a genotype, Max: highest value of a genotype. SD: standard deviation, CoV: Coefficient of variation.

Supplementary Tab 6 Average prediction accuracies from the three prediction approaches in the prediction scenario 1.

	**YLD**	**FLT**	**OIL**	**GSL**
GEBV-CV	0.74	0.99	0.76	0.86
GPA-CV	0.74	0.99	0.62	0.85
WFAM	0.20	0.49	0.47	0.27

Supplementary Tab 7 Average prediction accuracies from the four prediction approaches applied in the two subsets in the prediction scenario 2.

	**YLD**	**FLT**	**OIL**	**GSL**
**DH**				
GEBV-CV	0.69	0.68	0.82	0.88
GPA-CV	0.64	0.51	0.60	0.86
PPA-CV	0.63	0.49	0.58	0.85
WFAM	0.37	0.50	0.52	0.23
**SSD**				
GEBV-CV	0.39	0.72	0.72	0.60
GPA-CV	0.49	0.66	0.64	0.62
PPA-CV	0.49	0.65	0.62	0.62
WFAM	0.08	0.44	0.43	0.18
